# Demonstration for cold atmospheric pressure plasma jet operation and antibacterial action in microgravity

**DOI:** 10.1038/s41526-024-00408-1

**Published:** 2024-07-06

**Authors:** A. Rouillard, P. Escot Bocanegra, A. Stancampiano, S. Dozias, J. Lemaire, J. M. Pouvesle, E. Robert, F. Brulé-Morabito, M. Demasure, S. Rouquette

**Affiliations:** 1 GREMI, CNRS/Université d’Orléans-UMR7344, Orléans, France; 2 CBM, CNRS-UPR4301, Orléans, France; 3 Centre Hospitalier Universitaire d’Orléans, Orléans, France; 4 Centre National d’Études Spatiales, Paris, France

**Keywords:** Plasma physics, Microbiology

## Abstract

Cold atmospheric pressure plasma (ionized gas) is an innovative medical tool for the treatment of infected wounds thanks to its potential to inactivate drug-resistant microorganisms and promote tissue regeneration and vascularization. The low power consumption, compactness, and versatility of Cold Atmospheric Pressure Plasma (CAPP) devices make them an ideal tool for risk mitigation associated with human spaceflights. This work presents results in microgravity on the operability of CAPP and its antimicrobial effect. The experiments carried out in parabolic flights make it possible to optimize the treatment conditions (i.e., the distance, the gas mixture) and to obtain the rapid inactivation (<15 s) of *Escherichia coli* samples. Interestingly, the inactivation efficiency of CAPP was higher during parabolic flights than under terrestrial conditions. Overall, these results encourage the further development of CAPP medical devices for its implementation during human spaceflights.

## Introduction

Space exploration in the coming decades will require the preparation of long-duration human spaceflights. There are still many challenges to overcome in order to consider such expeditions in the near future. To anticipate these space journeys, it is essential to test innovative technologies that will allow people to live in full autonomy. Each mission aboard the International Space Station increases our knowledge of life in microgravity and ultimately allows us to predict what problems may arise during long periods in these special conditions. Biology and medicine hold a special place in the experiments carried out during the missions. The understanding of the effects of microgravity on the growth, virulence, and resistance of the microorganisms^1–3^, as well as the space lifestyle effect on the immune system^4^, are decisive for long-term missions in such environments. Risk mitigation associated with accidental acute wounds is of paramount importance in extreme conditions such as those encountered in space vehicles. Subject to weightlessness, confined, and almost isolated from the outside world, these places have limited volume and resources. Therefore, “space medicine” and “medication in space”, are part of expert groups focus of the THESEUS (Towards Human Exploration of Space—a European Strategy)^5^, covering health maintenance, the on-board medical resource management, the effects of spaceflight on pharmaco-dynamics or the unwanted side effects and toxicity of rugs medication in space. To minor microbial threats in the International Space Station, the air regeneration system is presently equipped with HEPA filters (high-efficiency particulate air), and surfaces are disinfected with quaternary ammonium or hydrogen peroxide agent, and infection are treated with antibiotics or antiseptic^6^. The Versatile, low-energy, and low-waste technologies are extremely valuable during these space missions. Technologies using cold plasma discharges appear definitively relevant and could likely be successfully adapted to these particular conditions for important applications such as the purification of air and wastewater, the disinfection of surfaces, biological supports, and human skin, the fight against drug-resistant bacteria and potentially pathogenic viruses^7–10^. Thus, knowledge of the characteristics and behavior of cold plasma in microgravity conditions is essential in the perspective of a strategic implementation of cold plasmas for future space missions, even if, until now, very few demonstrations of the use of the plasma in microgravity conditions have been reported^11^.

It is known that plasma can improve the treatment of patients with infected wounds by promoting vascularization, tissue oxygenation^12^ and regeneration^13^ and limiting infections by generating large concentration of Reactive Oxygen and Nitrogen Species (RONS), while keeping the overall gas temperature below 40 °C, allowing the plasma to be in direct contact with living tissues^14,15^.

This work targets to preliminary address terrestrial and spatial treatment of infected wounds with cold plasma. As a first step towards this challenging objective, a dedicated experiment was developed to study the performance of a plasma treatment, at room temperature and atmospheric pressure in air under microgravity conditions, of targets seeded with bacteria. These experiments could be carried out during three parabolic flight campaigns organized by the government agency responsible for France’s space policy in Europe (Centre National d’Etudes Spatiales: CNES). For this study, a plasma jet reactor device has been designed and developed since 2020 to operate during CNES parabolic flight campaigns (VP 164-62, VP 167-63, VP 171-64) linked to SAFE projects (pla**S**ma gun ther**A**py under zero-gravity parabolic **F**light conditions **E**xperiments) in 2022 and 2023. This device is based on the Plasma Gun® (PG) technology consisting of an electric generator that initiates gas discharge in a compact Dielectric Barrier Discharged (DBD) reactor^16^ and allows the delivery of cold atmospheric pressure plasma jet (CAPP) in ambient air in a gas volume or on dielectric or metallic targets. A specific rack composed of the PG device associated with different targets and diagnostic tools makes it possible to test cold plasma generation and bacteria exposure in microgravity conditions. Basically, this experiment consists of producing reactive species using the PG and assessing their effects in interaction with inoculated targets and is broken down into two parts documented in the result section:

- The first consists of generating a cold plasma by ionizing a flow of helium (pure or mixed with a few percent of oxygen) and delivering this latter in the air in order to determine the physicochemical properties of the plasma thus created. The geometric characteristics, using full frame and Intensified Charge Coupled Device (ICCD) cameras, will be studied according to the concentration of oxygen in the helium flow and to the presence or absence of gravity.
- The second allows the main objective of the study to test the action of plasma on an agar gel-type target loaded with bacteria. Time-dependent bacterial inactivation is investigated as a function of the distance from the target, the concentration of oxygen in the helium flow, and the presence or absence of gravity.

In the discussion, different analyzes based on additional laboratory experiments are presented in order to give insights on bacteria treatment processes in weightlessness.

## Results

### Plasma Gun operability and characterization

The first goal of this study was to demonstrate the PG operability in weightlessness conditions. The PG operated in the free expansion mode without any target downstream of the capillary outlet. Four different dioxygen admixtures with helium feed gas were tested: 0%, 0.5%, 1%, and 2%. The pulse repetition rate and voltage peak amplitude were set respectively at 20 kHz and +7 kV. Camera (conventional and ICCD) images were collected for the same operating conditions (DBD reactor, voltage peak amplitude, pulse repetition rate, and gas mixture) both during zero gravity flights (0 g, 825 hPa) and in the ground laboratory (1 g, 1013 hPa).

**Fig. 1:**
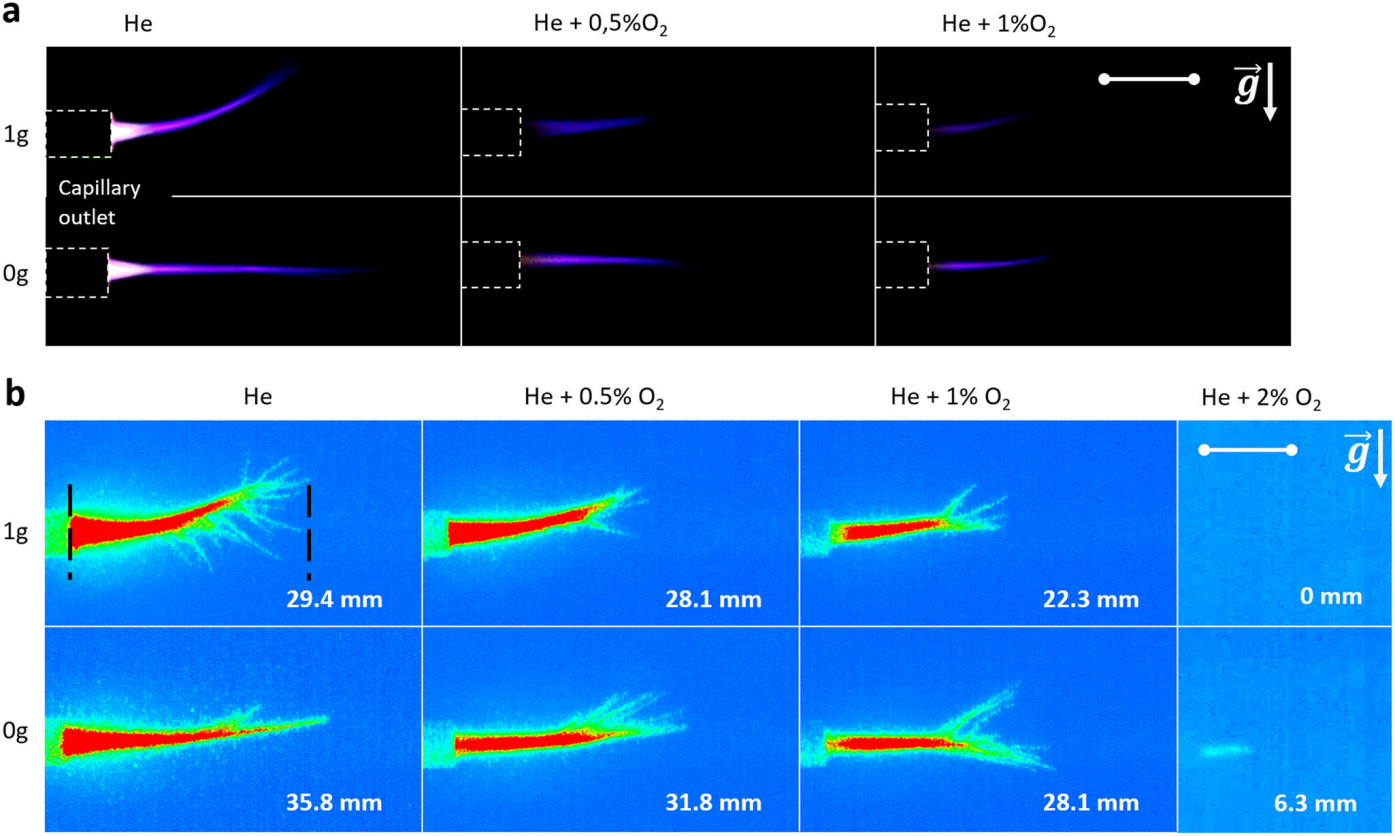
Plasma jet characterization in 0 g conditions. **a** Plasma jet camera photographs in 1 g and 0 g versus O_2_ concentration: 0%, 0.5%, 1%. Scale = 10 mm. **b** Plasma jet ICCD mean frame (30 frames) and mean length in 1 g and 0 g versus O_2_ concentration. Scale = 10 mm.

The Fig. 1a shows the plasma jet images at the capillary outlet captured in the visible wavelength range with the Canon 6D camera. For both gravity conditions, the plasma jet length decreases with the dioxygen concentration, and for 2% of O2, there is no visible plasma jet (data not shown). The main difference between the two gravity conditions is emphasized for helium only feeding gas. Indeed, in terrestrial conditions, the He buoyancy forces the plasma to develop itself upward^17^, which is not the case in 0 g condition.

This difference is due to the absence of buoyancy in weightlessness conditions. Dioxygen admixtures result in helium gas flow channeling when plasma discharge is ignited. This was reported in^18^ and attributed to the likely generation of large charged clusters in helium-dioxygen plasma jet operated in the PG configuration. The drift of these clusters during the voltage pulse application results in momentum transfer with ambient air molecules. So, for helium-dioxygen PG operation, the buoyancy impact is much less important, and the plasma plume tends to develop more horizontally. Nevertheless, in 0 g conditions, for any set of parameters, it appears from Fig. 1a that the plasma plume is longer and exhibits a different color pattern. This may be attributed to either microgravity or reduced pressure plasma jet operation. Work is ongoing to discriminate the role of these two parameters on plasma jet expansion.

Figure 1b displays the average of 30 single shots, 10 µs exposure, and ICCD images for 0%, 0.5%, 1%, and 2% dioxygen admixtures. All the images are displayed with the same contrast and brightness adjustments except for the 2% admixture, which is enhanced in that case. The ICCD images confirm the plasma jet generation collected with the conventional camera showing that the length of plasma propagation decreases as the admixture of O_2_ increases. We also observe the presence of an upward tilt in the plasma jet under 0 g conditions, though it is less pronounced for higher O_2_ concentrations, as explained previously. The relatively weak gas flow employed may be responsible for the extended stabilization time of the downstream gas flow and, consequently, for the observed plasma orientation. ICCD images were captured at the very beginning of the 0 g phase, just after the end of the 1.8 g phase. Furthermore, Fig. 1b illustrates differences in the plasma development. With He feed gas, we notice fewer branching structures along the plasma jet for microgravity conditions in comparison with the terrestrial conditions. This probably reveals the helium expansion with much less interplay with the surrounding air, as, during the microgravity phase, the shearing forces are dramatically minor. With dioxygen admixtures, branching structures do not appear along the plasma jet but mainly at its tip, where helium gas mixes with ambient air. The 2% O_2_ and He mixture images are obtained by enhancing the image contrast and brightness. However, under terrestrial conditions using the same scaling, the plasma jet is not visible. This work demonstrates that the plasma jet can be operated over a larger range of dioxygen admixtures 0 g conditions and that the plasma propagates over greater distances whatever the gas mixture compared to terrestrial conditions.

### Bacterial inactivation

**Fig. 2:**
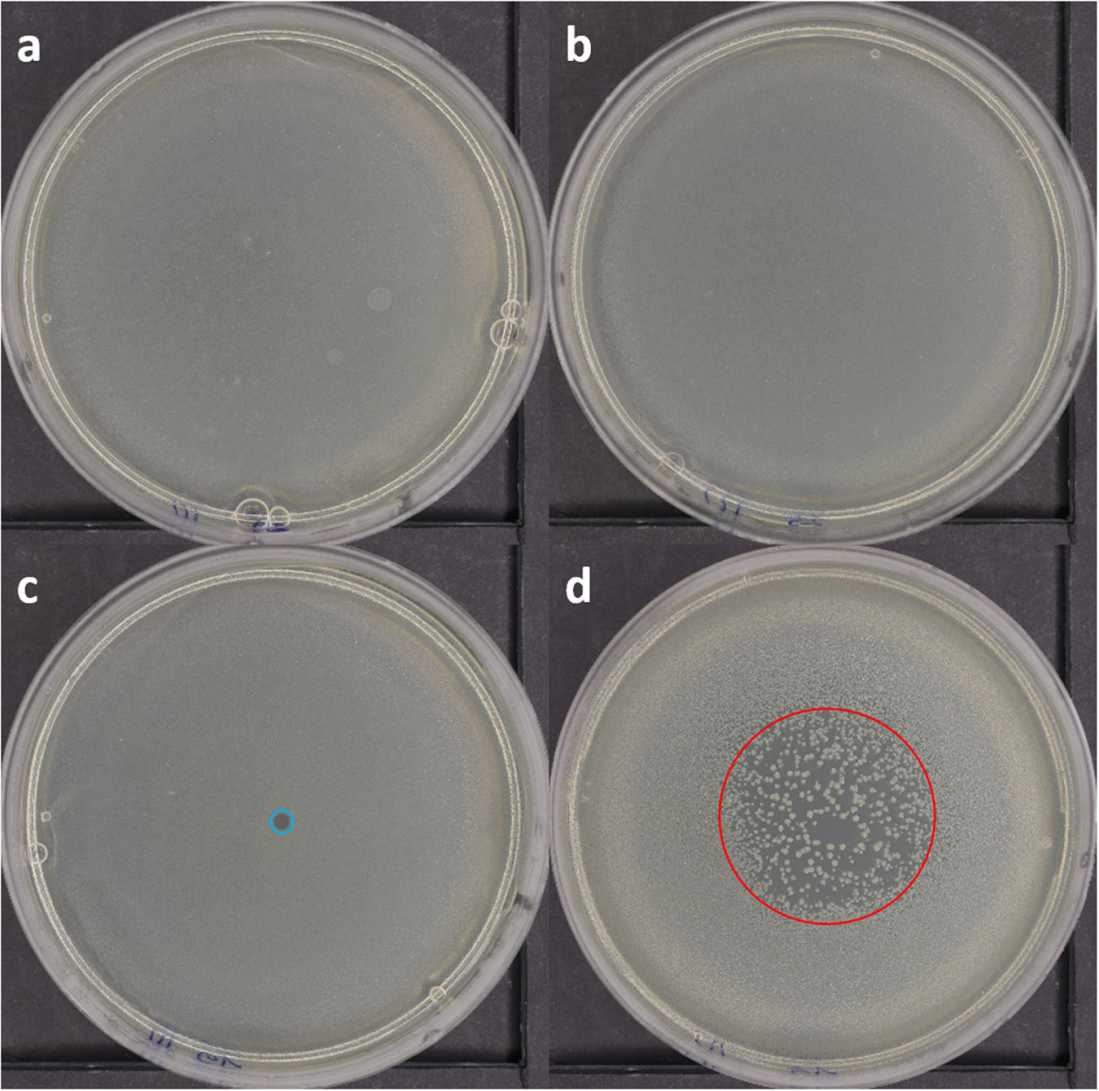
Inhibition of *E. coli* with the PG in 0 g conditions. **a** Untreated sample in terrestrial condition. **b** Untreated sample in weightlessness condition, exposed to a 5 min He gas flow. **c** Example of a fully inactivated inhibition area” (blue) after PG treatment (He, 7 kV, 20 kHz, 5 s). **d** Example of a non-homogeneously inactivated area (red) due to PG treatment, thereafter named ”effect area” (He + 2%O_2_, 7 kV, 20 kHz, 15 s).

Variations of the gravitational force and accelerations may influence the growth and metabolism of microorganisms or cells^1–3,19^. Anyway, In the experiment circumstances, the bacteria are subject to a total of 12 min in weightlessness and 35 min in 2 g, at 22 °C (the cabin temperature). During this amount of time and at this temperature, there could be 1 generation of *E. coli* at the most. Moreover, 0 g and 2 g phases are not in continue. Thus, the authors presumed it is not possible for the bacteria to be significantly mute because of the microgravity due to the short and fractioned exposure. As shown in Fig. 2, the control subjected to parabolic flights (31 cycles of hypergravity and weightlessness conditions) is identical to the control in standard terrestrial conditions developing in both cases a dense layer of bacteria colonies. The same is true for the controls, including exposure (5 min) to the He gas flow. This confirms that, for our experimental conditions, the parabolic flights, combined or not with He gas flow exposure, do not severely affect the E. coli growth. Conversely, samples treated by PG presented either small inhibition areas with well-defined boundaries and clear of any bacteria colonies (Fig. 2c) or larger non-fully cleaned and non-homogeneous areas (Fig. 2d), named here in the following as “effect areas”. Nevertheless, viability tests of bacteria sampled in the inactivated area and “effect” areas where no colony is observed after plasma exposure revealed the full inactivation of the bacteria in the two zones.

**Fig. 3:**
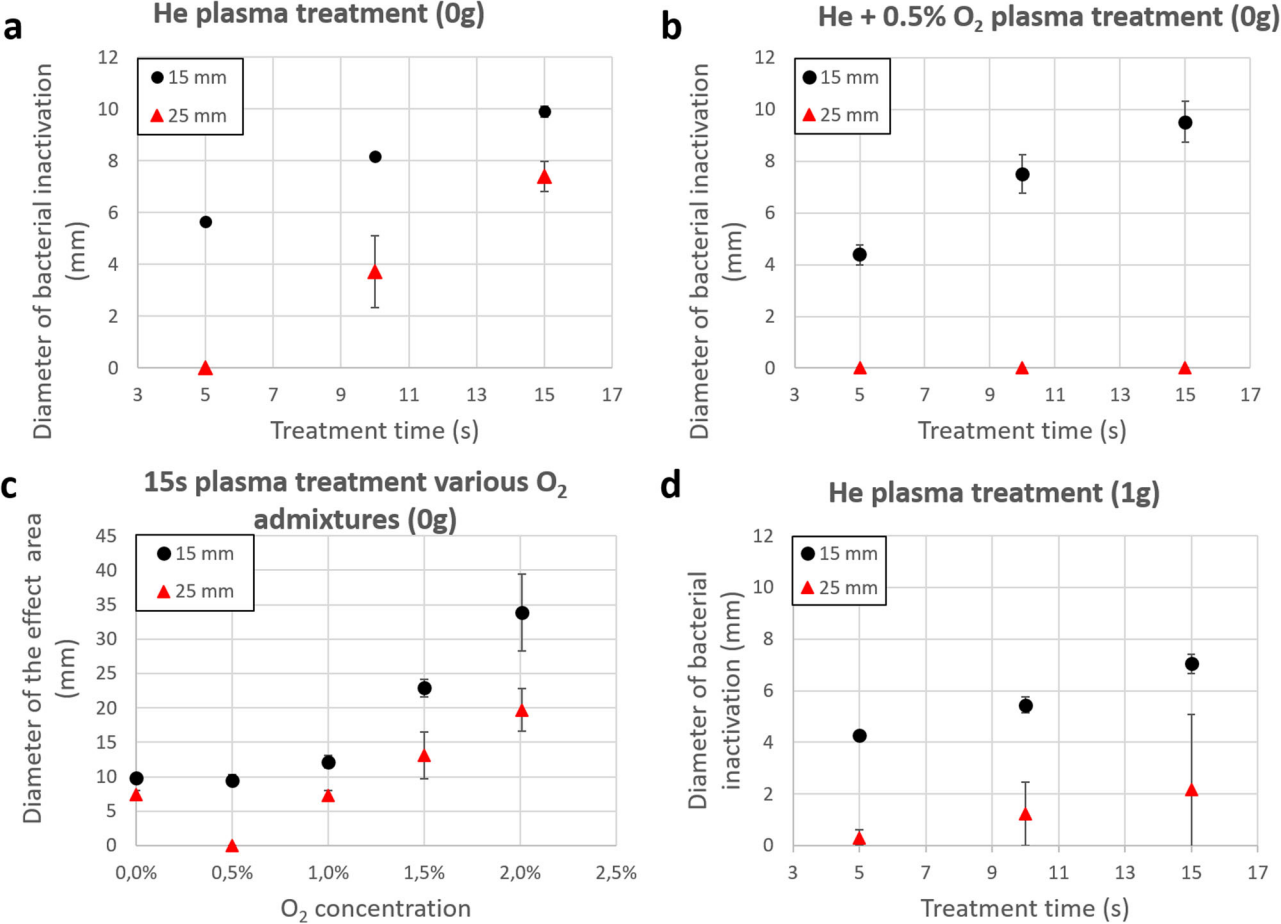
Bacterial inactivation analysis. **a** Diameter of bacterial inactivation zone versus treatment time following He plasma exposure at 20 kHz and 7 kV, for a reactor-target gap of 15 and 25 mm, in 0 g conditions. **b** Diameter of bacterial inactivation versus treatment time following a He–0.5% O_2_ plasma exposure at 20 kHz and 7 kV for a reactor-target gap of 15 and 25 mm in 0 g conditions. **c** Diameter of effect area following a 15 s-long He–O_2_ plasma exposure with different dioxygen admixtures at 20 kHz and 7 kV for a reactor-target gap of 15 and 25 mm in 0 g conditions. **d** Diameter of bacterial inactivation versus treatment time following He plasma exposure at 20 kHz and 7 kV for a reactor-target gap of 15 and 25 mm in terrestrial conditions (1 g). **a**–**d** Presented values are means ± SD of *n* = 4 biological replicates.

Figure 3a presents the diameter of the bacterial inhibition zone after a He plasma treatment, for two different reactor-target gaps, versus treatment time, in the weightlessness conditions. As expected, PG treatment reveals a time-dependent bactericidal action, as the diameter of inactivation increases with the treatment time. However, more surprisingly, even if the plasma plume is touching the target in both cases, the inactivation effect is very sensitive to the gap distance, especially for short treatment time.

Figure 3b shows the results obtained with the same set of experimental parameters but the gas mixture being in that case He + 0.5% O_2._ It exhibits a similar dependency on treatment time for a 15 mm target gap, although the measured inhibition diameters are slightly smaller compared to those with He-only feed gas. Conversely, there is no observed inhibition area for a gap distance of 25 mm.

In Fig. 3c, we report the effect area diameter for a 15 s treatment in 0 g condition, at 15 and 25 mm, for different admixtures, the O_2_ partial flow varying from 0% to 2%: 0%, 0.5%, 1%, and 2%. The diameter of the effect area was assessed from the determination of the boundary where the homogenous bacterial lawn had not grown in the Petri dish exposed to plasma.

Except for the 0.5% admixture, the diameter of the effect area gradually increases with the addition of dioxygen in the PG feeding gas for both gap distances. It corresponds to the shift between the fully inactivated area and the non-homogeneously inactivated area (Fig. 2).

Despite the similar diameter area between He–O_2_ 0.5% and He–O_2_ 1%, the inhibition effects are different. For 0.5% O_2_, the inhibition area is a fully inactivated area, whereas for 1% O_2_, it is a combination of a smaller fully inactivated area and a non-homogeneously inactivated area. The evidence for either inhibition or effect zones demonstrated for different operating parameters of the plasma jet implies different mechanisms of plasma action, as discussed thereafter.

## Discussions

First, we will focus on the inhibition area mechanisms encountered in our experimental conditions.

For a He gas plasma jet at 7 kV and 20 kHz, in weightlessness conditions (0 g), the plasma jet reaches the target for all studied gap distances and gas admixtures, according to ICCD acquisitions presented in Fig. 1b. The direct contact between plasma and the target could explain the clean inactivation area, by short-range effects most probably induced by short-lived reactive species, charged species, and UV. The plasma plume presents both in 0 g and 1 g a conical shape. Then, the longer the distance from the reactor, the smaller the section of the plasma plume and, therefore, the contact area with the target surface. This explains the reduced inactivation area for the wider gap (25 mm) compared to the smaller one (15 mm). The same is true for feeding gas containing O_2_ as, as this admixture shortens the plume length and leads to a smaller plasma-target cross-section and a smaller inhibition area. Inhibition areas obtained for the same operating conditions but in 1 g (Fig. 3d) present similar trends as in 0 g but with slightly reduced diameters. This is in agreement with the ICCD acquisitions that show a shorter plasma plume at 1 g compared to 0 g (Fig. 1b).

To further investigate the assumption that short-lived reactive species play a major role in determining the inhibition area, filtered ICCD acquisitions were performed to selectively visualize the location of some of these species. These short-lived species (O*, OH*, NO* in their excited states) are highly reactive and have strong oxidizing and bactericidal effect^20–22^. Having similar results for bacterial inactivation in both terrestrial and weightlessness conditions, the authors made the assumption that the inactivation mechanisms are similar in 1 g and 0 g. Therefore, the following ICCD analysis operated were all realized in terrestrial conditions. Acquisitions were done with an agar target during antibacterial experiments. However, the signal-to-noise ratio was rather weak, and the Petri dish side wall prevented an easy image collection, thus limiting the clear documentation of the data. While changing the target has an influence on the plasma features, we checked that similar emission patterns were observed with an agar plate or with a conductive target. Thus, ICCD-filtered imaging was performed on a flat grounded metallic target to enhance the emission intensity of the studied species transition.

**Fig. 4:**
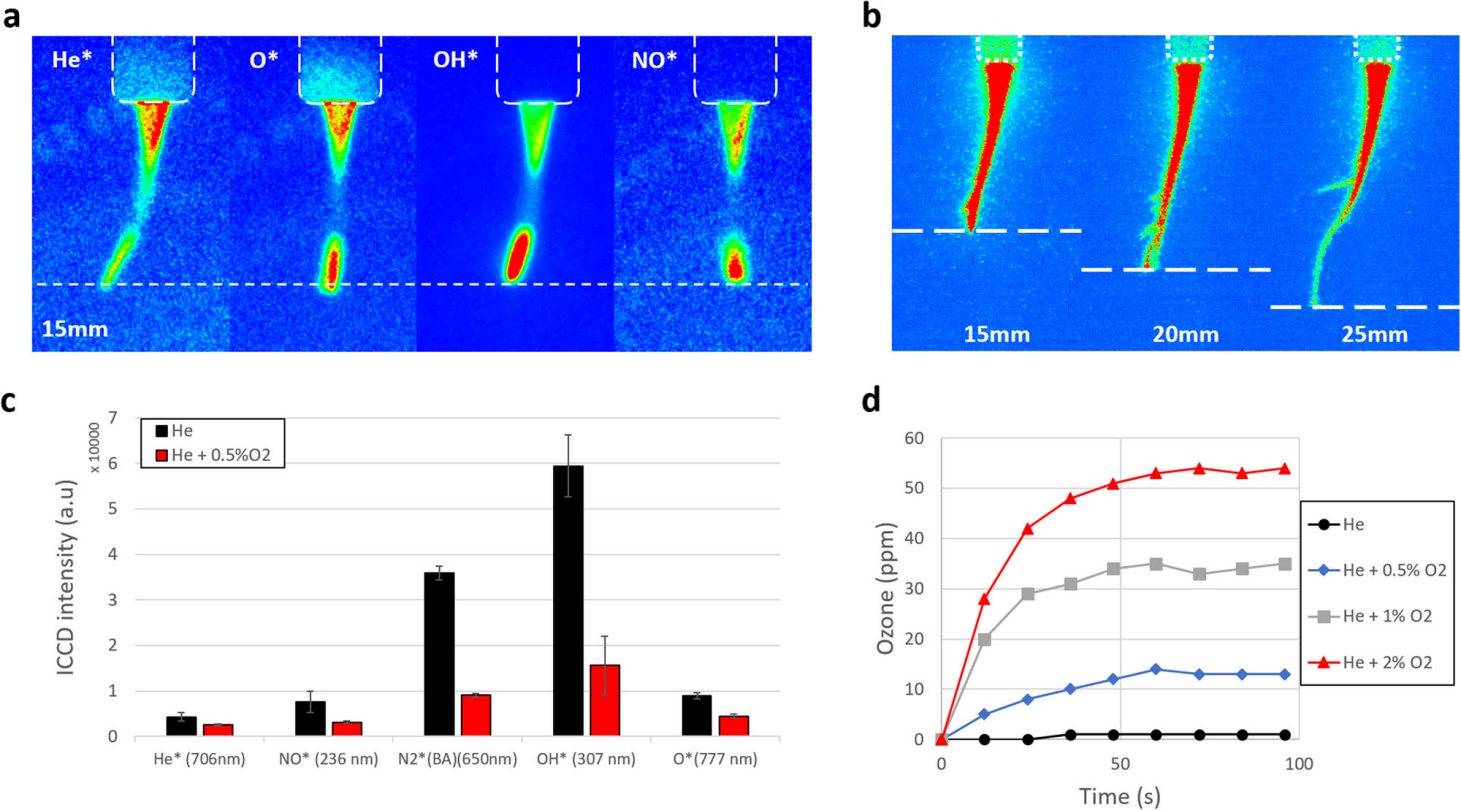
ICCD analysis and Ozone measurement in 1 g conditions. **a** Filtered ICCD acquisitions of a plasma treatment of a grounded target at 15 mm. Contrast and the brightness adjustment are optimally set up for each acquisition. **b** ICCD acquisitions of a 7 kV, 20 kHz He plasma treatment of a grounded target versus different reactor-target gaps: 15 mm, 20 mm, 25 mm. Contrast and brightness adjustment are the same for each acquisition. **c** ICCD intensities of different transitions at the surface of a 15 mm far grounded target for a He (black) and a He + 0.5% O_2_ (red) gas mixture for a plasma at 20 kHz and 7 kV. Presented values are means ± SD of *n* = 10 replicates. **d** Ozone measurement versus the O_2_ concentration at 20 kHz and 7 kV. The target is at 15 mm, metallic, and grounded. Presented values are means of *n* = 10 replicates.

As shown in Fig. 4a, the investigated short-lived RONS, O*, OH*, and NO* emissions are mainly created at the plasma-target interface. Note that the OH* filter is not selective enough to exclude any contribution from the nitrogen second positive bands at 315.9 nm, so the corresponding image in Fig. 4a is less demonstrative than that for atomic oxygen and nitric oxide emission patterns. Conversely, the helium emission pattern is much more intense at the tip of the PG capillary while Fig. 4a also evidence a second high-intensity zone close to the target. The strong population of the reactive species in the environment of the target results from the plasma jet impingement and counter propagation as previously discussed with grounded or floating potential targets^23,24^. Their huge reactivity implies that they will not diffuse far away from where they are produced but will play a major role in bacterial growth inhibition^25^.

As shown in Fig. 4b, larger gap distances lead to smaller plasma-target interfaces and so, less short-lived RONS are formed and enter in contact with the target.

The production of reactive oxygen species (ROS) could be enhanced by adding small percentage of dioxygen. Anyway, in our case O_2_ addition leads also to a shorter plasma plume and therefore of smaller area of contact. To quantify the impact of these two opposite effects of O_2_ addition to the RONS production, filtered ICCD acquisition intensity in proximity of the target for different O_2_ contents is documented in Fig. 4c.

Figure 4c reveals a reduction in He* production but also RONS production, whereas the dioxygen concentration increases. Thus, this suggests that, for the same PG configuration, the dioxygen addition in the gas medium quenches the O*, NO* (and N2*(B-A) that, by different reactions, creates NO*), OH*, O*, He* radicals’ production at the plasma-plume interface. It is confirmed by previous studies on the quenching effect of the dioxygen in He plasma^26^. With a pure He plasma jet, the VUV photons emitted by the He_2_^*^ promote the ionization of the dinitrogen and dioxygen at the plasma jet interface with the ambient air, inducing the RONS production. However, the admixture of dioxygen in the plasma consumed the He electrons energy in the O_2_ excitation and its dissociation^27^, decreasing the O_2_ and N_2_ ionization.

While adding more O_2_ reduces the inhibition area, at the same time it increases the effect area (Fig. 3b, c). Moreover, at 2%, the plasma plume does not reach the target, Fig. 1b, but the effect area diameter reaches its maximum (35 mm). This suggests that other, far-reaching mechanisms beyond those previously proposed are responsible for the observed effect area. Our assumption is that long-lived ROS, such as ozone (O_3_), play a major role. Ozone produced inside the plasma reactor can be transported in the gas flow and delivered to the target at considerable distances in a sufficient concentration to cause a bactericidal effect. This assumption is supported by the ozone concentration (Fig. 4d) measured at 2 mm of the plasma-target contact point (metallic grounded target) for different He and O_2_ mixtures: 0%, 0.5%, 1%, and 2%.

Figure 4d confirms that the addition of dioxygen in the feeding gas upstream of the reactor promotes ozone production in the downstream gas flow. As explained previously, the admixture of O_2_ in the plasma reactor favored the O_2_ dissociation.

1 $$e+{{\rm{O}}}_{2}\to 2{\rm{O}}+e$$

Thus, the oxygen atoms could form ozone through a tree-body reaction^28^

2 $${\rm{O}}+{{\rm{O}}}_{2}+M\to {{\rm{O}}}_{3}+M$$

(*M* could be O, O_2_, O_3_, He,…)

It is also worth noting that ozone production increases significantly between 0.5% and 1% dioxygen. Interestingly, there is already a notable ozone concentration at 0.5% after 15 s of operation, even though no effect area is visible in that case. Thus, the trends observed for the effect area in Fig. 3b and c could be partially explained by the concentration of O_3_ in the output gas flow. The minimum observed for 0.5% could be a consequence of a limited O_3_ production (compared to cases with a higher O_2_ initial content) and of a different fluid dynamic compared to the pure He case that limits the transfer of species to the target surface. Short-lived ones, mainly responsible for inactivation areas, are highly reactive and have a strong bactericidal effect. Long-lived species, such as ozone, are less reactive but more far-reaching. They can eventually achieve complete and clean inactivation but require longer treatment times. Anyway, the temporal limit imposed by the parabolic flights prevents the direct testing of this hypothesis in 0 g. Further investigations on this aspect are currently ongoing at 1 g at the GREMI laboratory. According to Directive 2008/50/EC of the European Parliament and of the Council of 21 May 2008^29^, the maximum permissible ozone exposure is 55 ppm for 8 h per day. The maximum ozone produced by the Plasma Gun is 54 ppm after 96 s. Although it is almost the maximum ozone production authorized, a plasma treatment lasts in the order of minutes. Moreover, the measurement was operated at the proximity of the plasma jet impinging spot. Therefore, there are no potential health threats due to the ozone exposure induced by the Plasma Gun treatment.

To conclude:

- In this study, the operability of a cold atmospheric pressure plasma jet in a weightless environment was successfully demonstrated.
- Secondly, The PG bactericidal efficiency in 0 g was characterized for different configurations. The inhibition efficiency of CAPP is higher under parabolic flight conditions than in 1 g.
- Further investigations put forward the inactivation mechanisms vary depending on the dioxygen concentration in the gas mixture and behave similar than in terrestrial conditions. The more dioxygen is adding, the smaller the inhibition zone is, but the larger the effect zone is. It shows the CAPP adaptability, optimizing O_2_ levels for precise control of the inactivation process, depending of the application.
- Further studies are currently underway to investigate the potential effects of the cabin reduced pressure. Moreover, new experiments are realized to increase the treatment time despite the short time allocated in 0 g condition.

Overall, this study not only showcases the adaptability of CAPP in a weightless environment but also provides insights into the diverse inactivation mechanisms and potential applications for decontamination in space. The CAPP opens promising implications for space exploration and healthcare in extraterrestrial confined environments.

## Methods

### Parabolic flights

**Fig. 5:**
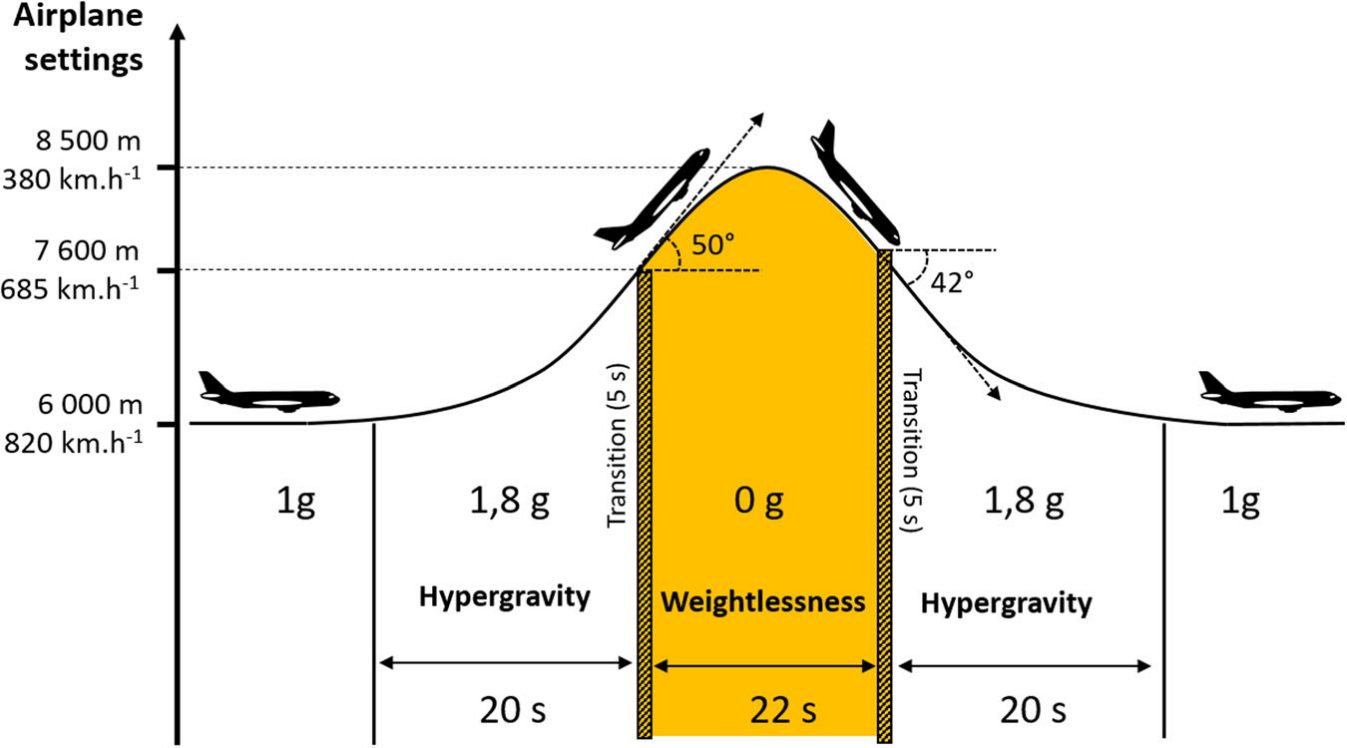
Parabolic flight. Airplane settings and gravity levels and duration during one parabola.

The experiments in microgravity are realized in an Airbus A310 air plane. The microgravity conditions are achieved during parabolic flight operation of the airplane^30,31^. A parabolic flight campaign organized by Novespace® lasts three days, including 31 parabolas each day. Each parabola is divided in three phases, and includes a ballistic trajectory, Fig. 5.

First, the plane accelerates and increases its pitch angle up to 50°. During this phase, objects and persons in the aircraft experience almost twice the gravity acceleration (1.8 g). Right after, the second phase is the weightless one and lasts 22 s. Finally, the plane returns to standard cruise conditions through a pull-out phase. During this phase, the felt acceleration is again almost twice the usual gravity. Additionally, during the flight, the cabin is pressurized at (825 ± 5 hPa, and the humidity is 15%^32^.

To resist to these rough environment conditions, an experimental rack was designed by the Groupe de Recherche sur l’Energétique des milieux ionisés (GREMI) in collaboration with Novespace® supporting team. The rack structure, the experiment design and the equipment fixations were calculated to endure 9 g emergency landing according to the Novespace® safety standards and restrictions^33^.

**Fig. 6:**
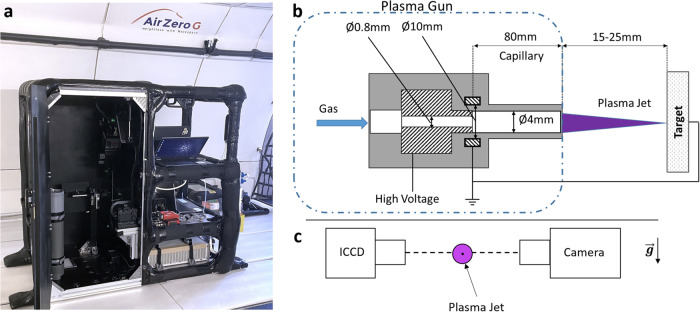
In-flight experimental setup. **a** In-plane photograph of GREMI experimental rack for cold plasma generation and bacteria exposure. **b** Plasma Gun reactor, side view. **c** Front view of the plasma jet and Optical setup.

The experimental rack basically consists of two zones, respectively documented on the left and right half parts of Fig. 6a. The right-hand side gathers the high voltage power supply used for plasma jet operation, gas flow meters, three computers used for gas flow, and two cameras control and a *Tektronix MDO3054* oscilloscope collecting voltage and current waveforms applied to the plasma jet reactor. The plasma jet reactor outlet (capillary) emerges in the left-hand side zone of the rack, this latter being where plasma jet delivery and real-time diagnostic are performed. Additionally, gas (helium and oxygen) bottles were set apart on the plane floor.

### Plasma source

In this work, the cold atmospheric plasma was generated in the PG reactor, and the so-called plasma jet was delivered outside the reactor in the ambient air. The plasma jet was characterized and used for bacteria exposure. The PG consists of a dielectric barrier discharge (DBD) reactor powered with microsecond duration high voltage pulses delivered in the kHz regime. DBD reactors have been deeply studied for their important RONS creation rate for bacterial decontamination and inactivation at atmospheric pressure^34^. In this work, the capillary DBD reactor was made of thermoplastic PEEK® (Polyether Ether Ketone, *RS PRO 514–770*) having a 100 mm length and inner/outer diameters of respectively 4/6 mm. The DBD reactor is equipped with an inner hollow (0.8 mm) high voltage powered electrode and a 10 mm wide grounded ring electrode set around the outer PEEK surface of the reactor. The tip of the inner electrode coincides with the grounded ring center, and the distance from the tip of the powered electrode to the dielectric capillary outlet, where the plasma jet emerges in ambient air, is 80 mm, as shown in Fig. 6b. The PG generator delivers 3 µs full-width half-maximum Gaussian shaped voltage pulses. Peak voltage amplitudes up to +7 kV and pulse repetition rates up to 20 kHz were applied to the DBD reactor high voltage electrode. In this study, the DBD reactor was flushed with pure helium or helium-dioxygen mixtures at a total gas flow rate of 1 slm. Helium (Air Liquide He N55) and helium-dioxygen (Air Liquide MC O_2_/He-UN1956) flows were monitored through two *Red-y GSC-B9KA-FF23 Vögtlin* flowmeters. The plasma jet was used following two different modes: (1) free jet expansion in ambient air and (2) jet application on the Petri dish target. During antibacterial experiments, the gap distance between the capillary outlet and the 90 mm in diameter Petri dish-contaminated surface was set to either 15 or 25 mm. The target was kept by a custom holder.

### Plasma diagnostics

The plasma jets were characterized by two cameras collecting images perpendicularly to the jet axis (Fig. 6c). The first one was a conventional Canon 6D camera with an aperture time of 1/50 s. The second one was an ICCD camera, model PIMAX3 Princeton Instrument. The ICCD camera was triggered at the onset of the high-voltage pulse delivered by the plasma generator. The acquisition mode was a repetitive gating mode with a gate width of 10 µs. ICCD measurements thus consist of time-integrated (during the full voltage pulse duration) single-shot snapshots. ICCD measurements were performed both during parabolic flights and on the ground in GREMI laboratory. In this latter case, the ICCD camera was also coupled with *Semrock* optical filters (Table 1) to observe the transitions of specific species in order to investigate their spatial distribution. In that case, the recordings were performed with a 50-voltage pulse averaging.

**Table 1:** Optical filters specification with the indication in the left column of the assigned excited species transitions

Monitored species	Center wavelength (nm)	Bandwidth (nm)
He*	711	25
N_2_* (B)	650	100
NO*	239	25
O*	777	12
OH*, N_2_*(C)	307	10

An ozone meter (*In-2000 locon* ozone analyzer) was used to measure ozone production. The ozone meter continuously sucks the gas through a flexible polytetrafluoroethylene tube with a flow rate of 1.5 slm to an absorption cell to quantify the ozone concentration. The tip of the sucking tube was set in close proximity to the plasma jet impinging spot over a metallic target.

### Bacterial sample inoculation protocol, plasma exposure, and analysis

The bacteria used are from an *E. coli* (CIP54 117) non-pathogenic and nonresistant strain provided by the Pasteur Institute. As pre-sowing protocol, bacteria are inoculated in 5 mL nutritive *LB broth (Lennox)* from *Condalab* to grow for 12 h at 37 °C in a shaking incubator to reach 10^8^ bacteria per mL. Liquid nutritive media for bacteria are sterilized in an autoclave then the *LB agar (Lennox)* from *Condalab* is dispensed in 90 mm diameter Petri dishes (*Sterilin*). 100 µL of the bacteria solution are inoculated in a 5 mL liquid medium.

The bacteria sowing was operated by the flooding method. The surplus was removed after 5 min. The Petri dishes were left to dry for 20 min in a laminar flow cabinet to dry for 20 min. Bacterial inoculation was processed about one hour before the plane took off each day. The plasma exposure was performed during the parabolic flight from 2 to 4 h after the flooding. Thus, in this work, the bacteria were treated during their exponential growth phase in order to have a qualitative measurement of the treated area, which is a standard antimicrobial susceptibility test^35–38^.

Following the plasma treatment and after the airplane landing, i.e., about one hour after the last plasma exposure during parabolas, the Petri dish was set for 12 h at 37 °C in an incubator.

Three types of controls were realized each day of the flight campaign. The first one was the bacteria growth control in the plane conditions, including all the gravity variations due to the parabolas. The second ones were the gas controls, corresponding to the Petri dish only exposed to the different gas mixture flow with no plasma. Then, they were compared to the third control, which consisted of the bacteria growth at the ground level.

Bacteria Petri dishes were stored in a tube dispenser, covered with a lid for the flight duration. They were set in a custom holder before the parabola (1 g phase), opened and exposed during the parabola, and covered and stored right after in the dispenser again. Each plasma treatment configuration (set parameters: voltage amplitude, frequency, He or He–O_2_ feeding gas, and treatment time) was duplicated four times in order to analyze at least triplicates. Thus, the presented values are means and standard deviations calculated from three experiments.

**Fig. 7:**
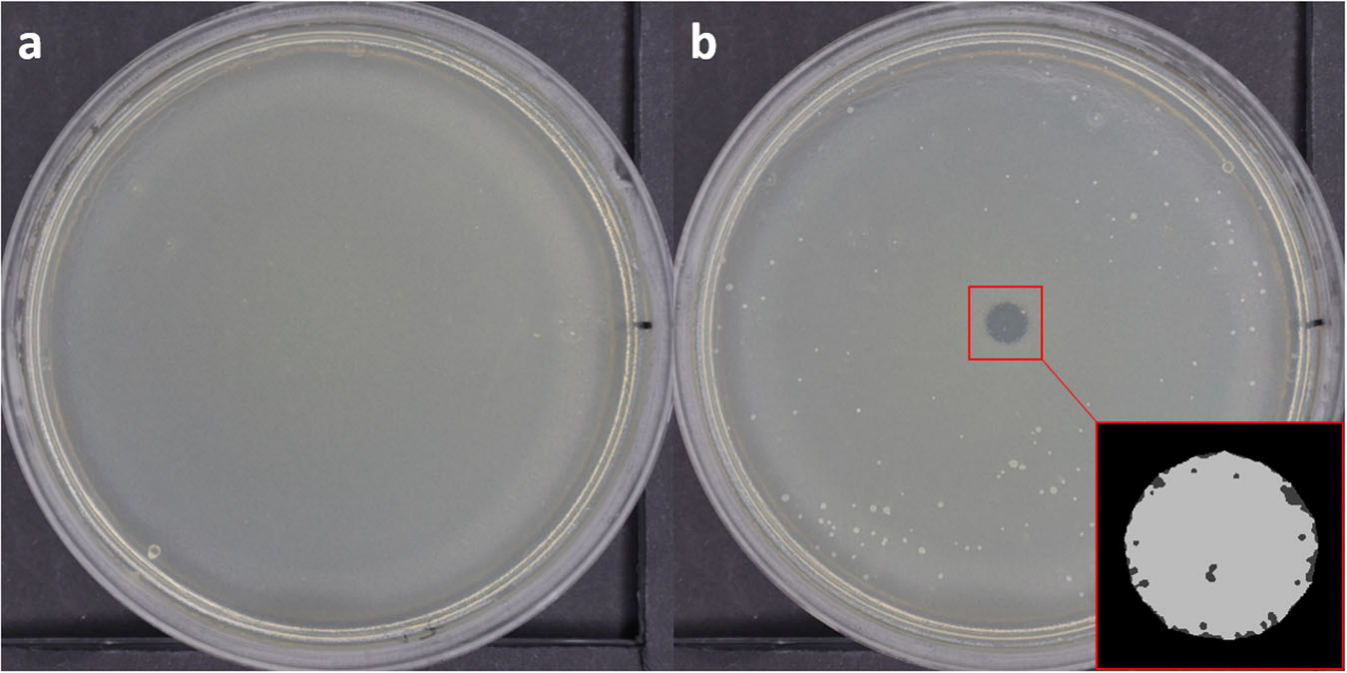
Bacterial sample analysis. **a** Control sample. **b** Photograph an example of a treated Petri dish with the result of image treatment to measure the treated area (right corner).

Once the colonies have grown, following the 12 h incubation, the Petri dishes are photographed with the Canon 6D camera. For control samples (exposed or not to gas flow in the plane or grown at ground level), the photograph exhibits a homogeneous whitish pattern resulting from the bacterial lawn growth over the 9 cm in diameter agar substrate (Fig. 7a). Conversely, for plasma treated samples, with induced bacterial inactivation, photographs show the appearance of more or less almost fully transparent circular zones on the images as shown in Fig. 7b. These photographs were processed thanks to a MATLAB imaging program to calculate the diameter of either inhibition or effect zone following the plasma exposure.

## Data Availability

All relevant data are avaible from the corresponding author on request.
